# Relationship between Key Environmental Factors and the Architecture of Fruit Shape and Size in Near-Isogenic Lines of Cucumber (*Cucumis sativus* L.)

**DOI:** 10.3390/ijms232214033

**Published:** 2022-11-14

**Authors:** Tingting Zhang, Yuanyuan Hong, Xuan Zhang, Xin Yuan, Shuxia Chen

**Affiliations:** 1 College of Horticulture, Northwest A&F University, Xianyang 712100, China; 2 Shaanxi Engineering Research Center for Vegetables, Xianyang 712100, China

**Keywords:** cucumber, fruit shape and size, fruit cell, thermal effectiveness, photosynthetic active radiation, logistic regression

## Abstract

Fruit shape and size are complex traits influenced by numerous factors, especially genetics and environment factors. To explore the mechanism of fruit shape and size development in cucumber, a pair of near-isogenic lines (NIL) *Ln35* and *Ln37* were used. The fruit length and diameter, cell length and diameter, and related gene expression were measured. Both the fruit length, diameter, and cell length and diameter showed sigmate curves in the two lines. The cell length and diameter were significantly positively correlated with fruit length and diameter both in two lines. The expression of *CsACS2* and *CsLNG* showed significant positive correlations with fruit length and diameter increment in *Ln35*, and there was no correlation in *Ln37*. Furthermore, there were significant positive correlations between fruit size and thermal effectiveness (TE), as well as between fruit size and photosynthetic active radiation (PAR), both in two lines. Two models using logistic regression were formulated to assess the relationships among fruit length and diameter in *Ln35* and *Ln37*, respectively, based on thermal effectiveness and photosynthetic active radiation (TEP). The coefficient *R*^2^ values of the models were 0.977 and 0.976 in *Ln35*, and 0.987 and 0.981 in *Ln37*, respectively. The root mean square error (RMSE) was 12.012 mm and 4.338 mm in *Ln35*, and 5.17 mm and 7.082 mm in *Ln37*, respectively, which illustrated the accurate and efficient of these models. These biologically interpreted parameters will provide precision management for monitoring fruit growth and forecasting the time of harvesting under different temperatures and light conditions.

## 1. Introduction

In recent years, fruity vegetable production in greenhouses has played a very important role in the development of the regional economy. Fruit is the main product of fruity vegetables, and it is also an important factor in appealing to consumers. Consumer preferences were determined greatly by the fruit appearance quality to some extent [1]. The cucumber (*Cucumis sativus* L.) is one of the most important fruity vegetables mainly cultivated in the greenhouse, and diversification of fruit shape can meet the requirement of consumers to more varieties and specialization with the improvement of living standards; in addition, the appropriate size of fruits will add additional value of products [2]. However, fruit shape and size were greatly influenced by numerous factors, especially temperature, light, and humidity conditions [3].

During cucumber fruit development, the range of cell expansion determined the final cell size of the fruits, which was considered as the major determinant factor for fruit size [4], while the cell number also exhibited a positive correlation with the growth of fruit length and diameter [5,6,7]. Both cell size and number altered the fruit shape and size in cucumber [8,9]. However, the mechanism that determined the fruit cell number and size was still unclear. Related genes that participated in the regulation of cucumber fruit shape and size were considered important factors. *CsLNG* was a homologous gene of *LONGIFOLIA1 (LNG1*), which regulated slender leaves in *Arabidopsis*, and was considered as the candidate gene of cucumber fruit length QTL loci *FS2.1* [10,11]. Researchers reported that the *CsLNG* regulated cucumber fruit length by controlling the cell numbers using NILs with *CsLNG* genetic background [4]. *CsSUN* gene encoded IQ-domain protein of *IQD* gene family controlled cucumber fruit length growth by influenced the fruit cell numbers [4,10]. Enzymes, transcription factors and proteins were reported to involve in the regulation of fruit size [12]. For example, dysfunction of *CsACS2* reduced ethylene production and repressed cell division, and resulted in shorter fruit in cucumbers [13]. Knockdown of the *CsFUL1^A^* transcription factor caused further fruit elongation and larger cell size than in wide-type lines [8]. Furthermore, *CsSUP*, which was regulated by *CsFUL1^A^*, participated in the reduction in fruit length and smaller and disorganized cells compared with the wide-type line [8]. *CsFNL7.1*, which encoded a late embryogenesis abundant protein, was considered to control cucumber fruit neck length via modulated fruit cell expansion [4]. There were some major gene loci to regulate the formation of cucumber fruit shape together with some others [14,15,16,17]. 

Environmental factors were the main external factors that affected fruit shape and size the most. It was believed that temperature and light were two important factors in fruit development [18]. The key environmental such as temperature, light intensity, and irrigation played important roles in fruit growth [19]. Fruit expansion rates of apples were affected by the daily temperatures, and smaller fruit were produced in low-temperature conditions [20]. The largest pepper fruits were obtained under optimal light and temperature conditions, and small fruits were obtained at low temperatures [21]. Higher fresh weight was obtained in high light intensities conditions for tomatoes [22,23]. Supplemental lighting, as an important light-controlling technology, was widely used in the production of fruity vegetables under the protection structure [24,25]. In addition, obviously, solar radiation and temperature played important roles in cucumber fruit architecture, development, and fruit shape. It is essential to regulate environmental factors to form better fruit size and shape to increase the additional value of products [26]. However, it was unclear whether to regulate fruit shape and size through precision management of environmental factors, especially the most important factors, temperature and light. Our findings suggest that the proposed workflow aids in the identification of key metabolites in the central metabolism that responds to monochromatic red-LED treatment and contributes to increasing the fruit size of tomato plants.

The logistic regression method was helpful to understand the development of fruit size and shape [27,28]. Models of effective fruit growth benefited the upgrades of management of key environmental factors [29]. The effects of temperature and light on the architecture of fruit shape were always comprehensive. However, the simulation models established were mainly focused on a single environment, such as light or temperature [30,31]. Recently, thermal effectiveness and photosynthetic active radiation (TEP) were considered as comprehensive parameters that played the important roles in the development of crops [32,33]. The TEP-based models provided a more accurate method to forecast the dynamics of plant growth [32].

In order to explore a clearer mechanism of regulation in cucumber fruit architecture, provide precision management, and exploit the full potentials quality of certain cultivars, the relationship among the architecture of fruit size, cell size, gene expressions, and the environmental factors in cucumber near-isogenic lines (NIL) were probed, logistic regression between fruit size and temperature and light were analyzed. It will provide some basis for precise management through the management of temperature and light. 

## 2. Results

### 2.1. Changes in the Key Environmental Factors in the Plastic Tunnel

**Figure 1:**
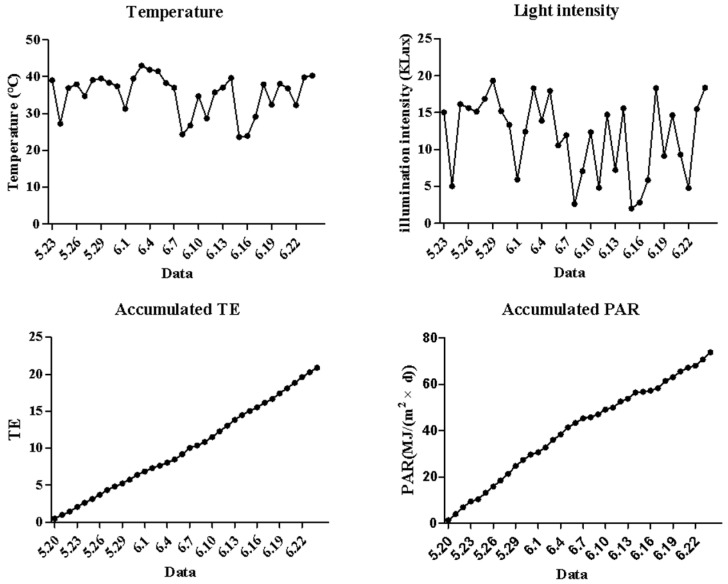
Changes in temperature and light intensity in the plastic tunnel.

The environmental temperature and light intensity in the plastic tunnel were showed in Figure 1. The average daily light intensity ranged from 3.52 Klux to 38.40 Klux, increased from 26 May to 31 May, and decreased from 4 June to 15 June. The average daily air temperature ranged from 22.45 °C to 27.97 °C. The highest temperature appeared at 14:00 o’clock on 3 May, which is 43.25 °C. The lowest temperature appeared on 28 May 2020 at nighttime and was 11.55 °C. Both the accumulation of RTE and accumulation of PAR showed linear patterns from 23 May to 24 June 2020.

### 2.2. Changes in Fruit Shape and Size during the Fruit Development

**Figure 2:**
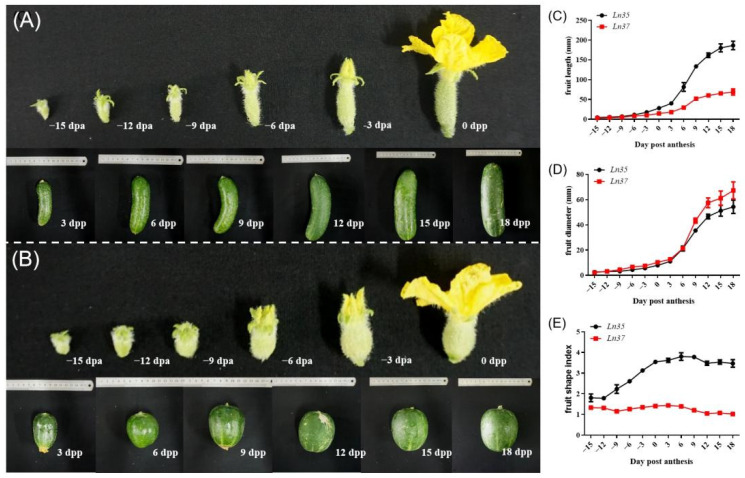
Changes of fruit shape during fruit development in two NILs lines. (**A**,**B**) is the dynamic changes of fruit in *Ln35* and *Ln37*, respectively. (**C**–**E**) was the dynamic changes in fruit length, fruit diameter and fruit shape index in the two lines.

The changes in fruit length and diameter of *Ln35* and *Ln37* were measured, and the fruit shape index was calculated (Figure 2). It showed that the most significant difference in fruit shape between *Ln35* and *Ln37* was observed at 3 dpa (Figure 2A,B). The growth dynamics of fruit length in both *Ln35* and *Ln37* showed sigmate curves, and it increased rapidly from 3 dpa (Figure 2C), and then the increase in fruit length in *Ln35* was faster than that in *Ln37*, and the length of *Ln35* was 1.94~2.58 times of *Ln37* from 0 dpa to 18 dpa, which represented the ovary stage, picked stage and mature stages. The growth dynamics of fruit diameter in both *Ln35* and *Ln37* also showed sigmate curves (Figure 2D), and there were no significant differences between the fruit diameter of both *Ln35* and *Ln37* before 6 dpa. The fruit diameter of *Ln37* had a rapid increase at 6 dpa, and it was significantly larger than that in *Ln35* from that time on. There was also a significant difference in fruit shape index between the two lines from −12 dpa to 18 dpa. The fruit shape index of *Ln35* ranged from 1.78 to 3.78 and the fruit shape index of *Ln37* ranged from 1.02 to 1.44.

### 2.3. Changes of Fruit Cell Shape in NILs

**Figure 3:**
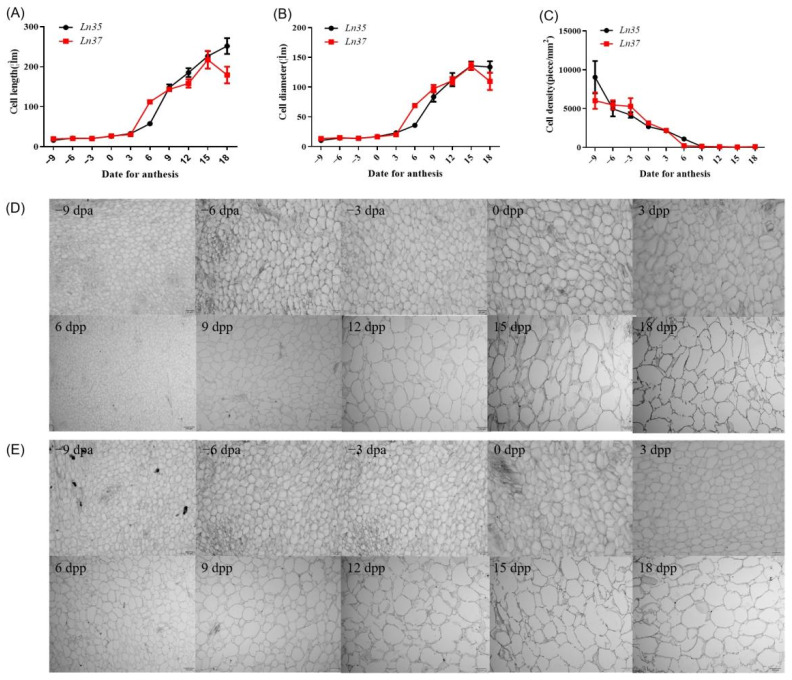
Fruit mesocarp cells shape in NILs. (**A**–**C**) are the changes in cell length, cell diameter, and cell density in NILs, respectively. (**D**) is the dynamic cellular changes of fruit cells in *Ln35*, (**E**) is the dynamic cellular changes of fruit cells in *Ln37*.

The fruit cell length, diameter, and cell density of *Ln35* and *Ln37* were observed and showed in Figure 3. The growth dynamics of cell length and cell diameter in the fruit of *Ln35* and *Ln37* showed sigmate curves and it increased rapidly from the 3 dpa (Figure 3A,B). From then on, the fruit cell length and diameter increased rapidly before 18 dpa. The cell length of *Ln35* at 18 dpa was 7.62 times of that at 3 dpa, the cell length of *Ln37* at 18 dpa was 5.89 times of that at 3 dpa, the cell diameter of *Ln35* at 18 dpa was 5.78 times of that at 3 dpa, and the cell diameter of *Ln37* at 18 dpa was 5.45 times of that at 3 dpa, indicating that the cell length increased more fastly in *Ln35* than that in *Ln37* during this period, whereas the cell diameter increased similarly in both lines. 

There was a significant difference in cell size between the two lines from 6 dpa to 18 dpa. At 6 dpa, the cell length and diameter of *Ln37* fruit were significantly longer than that of *Ln95*. At 18 dpa, the fruit of *Ln97* had a decreasing trend, which resulted in shorter cell length and diameter than that of *Ln95*. The cell density in *Ln35* and *Ln37* decreased rapidly from −15 to 6 dpa, while slowly decreasing were observed from 9 to 18 dpa. The rapidly increasing of fruit length and diameter from 3 to 12 dpa was mainly caused by both the rapidly increasing of the cell length and diameter, which implied that cell expansion plays important role in fruit size changes from 3 to 12 dpa.

### 2.4. The Relative Expression of Genes Related to Fruit Shape 

**Figure 4:**
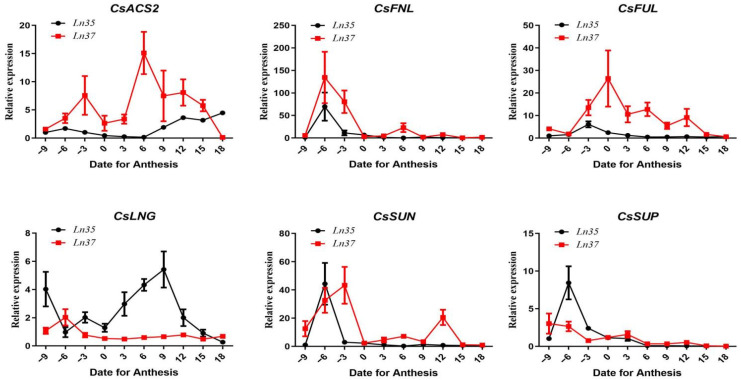
The relative expression of fruit shape genes in fruit development.

The gene expressions of *CsSUN*, *CsFNL*, *CsSUP*, *CsACS2*, *CsFUL*, and *CsLNG* were examined during fruit development in *Ln35* and *Ln37* (Figure 4). *CsSUN*, *CsFNL*, and *CsSUP* had similar expression patterns in both lines during fruit development. They exhibited a high level at pre-anthesis stages and decreased to a low level after 0 dpa, except that the *CsSUN* gene had a higher expression level at 12 dpa in *Ln37* than that in *Ln35*. Whereas *CsACS2*, *CsFUL*, and *CsLNG* had different expression patterns in two lines. The expression of *CsACS2* peaked at 6 dpa in *Ln37*, while it had a rather low expression level in *Ln35*. The expression pattern of *CsFUL* was similar to that of *CsACS2*, which peaked before 0 dpa in *Ln37*, while its expression was rather low in *Ln35*. The *CsLNG* gene had a significantly high expression level in *Ln35*, while it had a rather low expression level and exhibited little change in *Ln37*. The expression level of *CsLNG* increased rapidly from 0 dpa to 9 dpa in *Ln35* and peaked at 9 dpa, which was 8.2 times *Ln37* at 9 dpa. The difference in *CsLNG* expression in two lines indicated that *CsLNG* might play an important role in the increment of fruit length of *Ln35*.

### 2.5. The Relationship between Cell Size and Fruit Shape and Size

The relationships between the cell and fruit of *Ln35* and *Ln37*, respectively, were analyzed (Table 1). There were strong positive correlations between cell length and fruit length, cell diameter, and fruit diameter in both two lines, indicating that cell size influenced the fruit size in cucumbers. 

The cell density value was determined by cell size and cell number. However, the fruit shape index had negative correlations with cell length and cell diameter in *Ln37*, and it had no significant correlation in *Ln35*. Moreover, the fruit shape index was negatively correlated with cell density in *Ln35* and no significant correlation in *Ln37*. These results implied that the cell number might contribute to the difference in fruit shape index between *Ln35* and *Ln37*.

### 2.6. The Relationship between Gene Expression and Fruit Increment

The relationships between fruit shape increment and gene expression and fruit cell increment and gene expression in both lines were analyzed (Table 1). There was a significant positive correlation between fruit *CsLNG* expression and fruit length increment in *Ln35*, and it had no significant correlation in *Ln37*. As well as, *CsACS2* expression significantly positively correlated with fruit length increment and fruit diameter increment in *Ln35*, and it had no significant correlation in *Ln37*. The other four genes, *CsFLU*, *CsSUN*, *CsFNL*, and *CsSUP*, showed no significant correlations in both two lines. In addition, *CsLNG* expression significantly positively correlated with cell diameter increment in *Ln35*, and it had no significant correlation in *Ln37*. *CsACS2* expression is significantly positively correlated with cell length increase in *Ln37*. *CsFLU*, *CsSUN*, *CsFNL*, and *CsSUP* showed no significant correlations with cell increment in both two lines. These results indicated that *CsLNG* and *CsACS2* genes might be hereditary factors for the difference in cell size and fruit size between *Ln35* and *Ln37*. *CsLNG* and *CsACS2* contributed to fruit elongation by affecting the cell size.

### 2.7. The Correlation between Key Environmental Factors and Fruit Shape and Size Increment

For fruit length and diameter, which were the cumulative variables and cannot be explored, the relationship with environmental factors at one time point. Thus we analyze the relationship between the product of thermal effectiveness (TE) and fruit shape and size traits and between the photosynthetic active radiation (PAR) and fruit shape and size traits. The results are exhibited in Table 2. Both the TE and PAR were significantly positively correlated with fruit length and fruit diameter in the *Ln35* and *Ln35*. The results indicate that the TE and PAR values influenced the fruit size.

Two-way analysis of variance (ANOVA, Table S2) was used to determine the effect of cucumber materials and seasons on fruit length, diameter, and fruit shape index. There were no significant interactions found between cucumber materials and seasons on fruit length and fruit diameter. *Ln35* has a significantly longer fruit length and a larger fruit shape index value than *Ln37*. However, there was significant interaction found between cucumber materials and seasons on the fruit shape index. Furthermore, there was no significant effect of season on fruit length, diameter, and fruit shape index. These results suggest that the autumn of 2020 fruit shape data can be used to validate the models that model in the spring of 2020. Then, there were significant differences in fruit shape development between *Ln35* and *Ln37*, which need to be represented by different fruit shape development models.

### 2.8. Logistic Regression of the Fruit Shape Based on TEP

To probe the relationship between fruit shape traits and environment, the product of thermal effectiveness and photosynthetic active radiation (TEP) value was adopted to probe the relationship of light and temperature with cucumber fruit length, diameter, and volume.

**Figure 5:**
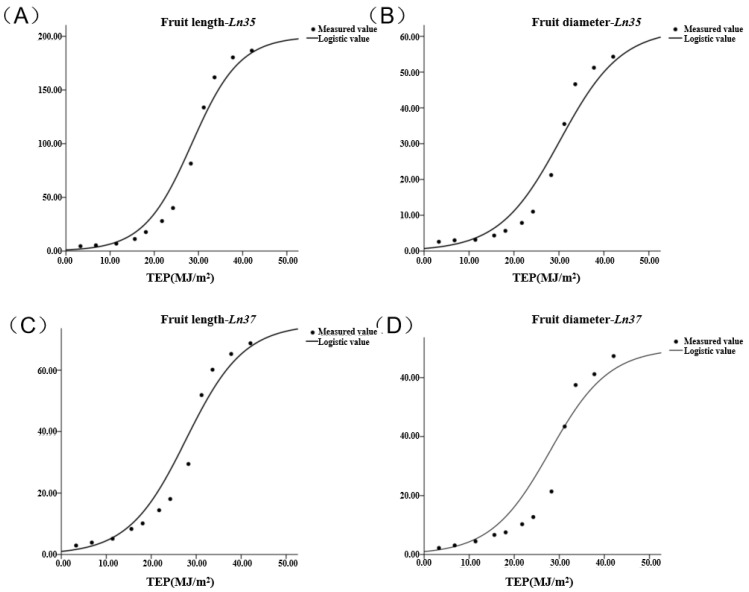
Relationship between fruit shape and TEP under *Ln35* and *Ln37*. (**A**) is the model of fruit length in *Ln35*; (**B**) is the model of fruit diameter in *Ln35*; (**C**) is the model of fruit length in *Ln37*; (**D**) is the model of fruit diameter in *Ln37*.

The logistic equation was performed using Formulas (1)–(4). The results are shown in Figure 5 and Table 3. The fruit length and diameter increased slowly when the TEP was lower than 20 MJ/m^2^ in both *Ln35* and *Ln37*. Fruit length increased rapidly when the TEP was between 20 MJ/m^2^ and 40 MJ/m^2^. Both *Ln35* and *Ln37* had maximum growth rates when the TEP was about 31.2 MJ/m^2^. However, there were significant differences between fruit length in the two lines. *Ln35* increased by 17.42 mm per day, while *Ln37* increased by 7.46 mm per day at the maximum growth rate time. The TEP accumulated from 20 MJ/m^2^ to 40 MJ/m^2^, and the fruit length increased by 143.68 mm in *Ln35* and 49.31 mm in *Ln37*. When TEP was higher than 40, the growth speed of fruit length slowed down.

Fruit diameter increased rapidly when the TEP was between 20 MJ/m^2^ and 42 MJ/m^2^ in both lines. Similar to fruit length, the maximum growth rate of fruit diameter is the time when TEP was accumulated to 31.2 MJ/m^2^ in both *Ln35* and *Ln37*. At this time, *Ln35* increased by 4.76 mm per day, while *Ln37* increased by 7.34 mm per day. The TEP accumulated from 20 MJ/m^2^ to 42 MJ/m^2^, and the fruit diameter increased to 41.80 mm in *Ln35* and 47.57 mm in *Ln37*. When TEP was higher than 42 MJ/m^2^, the growth speed of fruit diameter slowed down both in *Ln35* and *Ln37*. 

*R*^2^ and RMSE were tested to determine if these models were suitable or not for the growth of fruit shape in *Ln35* and *Ln37*. It showed that the *R*^2^ of fruit length and fruit diameter were 0.977 and 0.960 in *Ln35*, and 0.976 and 0.987 in *Ln37*, respectively. RMSE was 12.012 mm and 4.338 mm in *Ln35*, and 5.17 mm and 7.082 mm in *Ln37*, respectively. It indicated that the models of fruit length and diameter based on the TEP in both *Ln35* and *Ln37* were accurate and efficient (Table 3). These models can provide an accurate harvesting time, which is helpful information for productive behavior.

### 2.9. Validation of the Model

**Figure 6:**
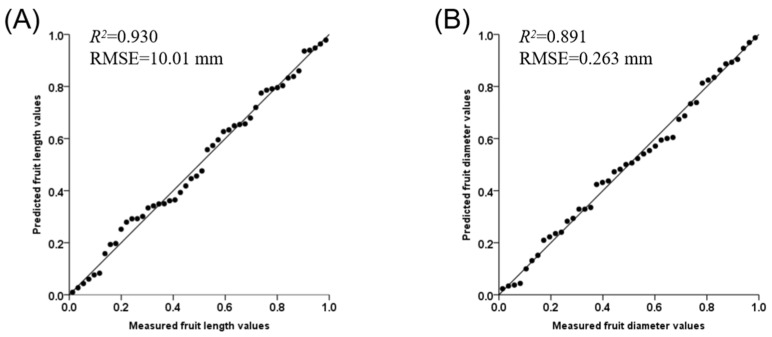
Comparison between predicted and measured values of cucumber fruit length (**A**) and diameter (**B**).

The data of fruit length and diameter of *Ln35* and TEP during fruit development were collected in 2020 autumn to verify the models (Table 1). It was found that the determination coefficient *R*^2^ was 0.930 and 0.891 in fruit length and diameter, respectively (Figure 6). The value of RMSE in fruit length and diameter during development in 2020 autumn was 10.01 mm and 0.263 mm, which meant suitable conformity between predicated value and measured values in fruit length and diameter, and these models of fruit shape traits were reasonable representations for the actual system. 

## 3. Discussion

### 3.1. Cell Division and Expansion in Fruit Shape and Size Morphogenesis

The appearance quality of cucumber fruit not only enhanced the aesthetic degree but also improved its commodity values. Previous studies have been carried out on various cucumber lines for fruit and cell growth [5,6,7,34]. These results showed that cell division occurs before anthesis and continues for about 3-6 days after anthesis in cucumber fruit, remaining more or less constant in further growth. Cell expansion occurs after cell division or at the end of cell division stages and with rapid fruit growth [5,6,34]. However, many of the prior studies on cucumber fruit development have examined growth post pollination or the ovaries’ approach to anthesis [35,36]. Here, we explored the morphological and cellular changes during fruit development, which ranged from −15 dpa to 18 dpa in two *CsLNG* cucumber NILs. Consistent with earlier work [5,6], cell size had changed little before 3 dpa, implying the fruit length increased by cell division at the early stages. The peak of fruit length and diameter increase in *Ln35* and *Ln37* coincided with the peak of cell expansion from 3 dpa to 15 dpa, indicating that cell expansion significantly promoted the fruit size increase in the two lines. There was no difference in fruit cell size and shape between *Ln35* and *Ln37* before the 6 dpa point. However, a greater difference in fruit length and fruit shape index between *Ln35* and *Ln37*, and the difference became apparent at −15 dpa. These results implied that the differences in fruit length and shape index before 6 dpa in two lines were influenced by cell division. After 6 dpa, it exhibited a consistently greater difference between *Ln35* and *Ln37* in fruit diameter and cell shape index. *Ln35* fruits diameter and cell shape index were larger than *Ln37*. It revealed that the difference of NILs in fruit diameter after 6 dpa was influenced by fruit cell shape. 

### 3.2. Effects of Genetic and Environmental Factors on Cucumber Fruit Development

The related gene expression can determine the development of fruit appearance quality, especially for fruit shape and size [37]. In cucumber fruit development, the related genes that participated in the regulation of fruit shape and size have been reported for many years [4,10,11,12]. However, the development of fruit shape and size was regulated by many different genes for different cucumber germplasms. 

Environmental conditions would affect the growth of any plant organ under which it develops [38]. The environmental conditions were generally considered temperature regimes, light availability, soil characteristics, water availability, and so on. There have many plant organ growth influenced by the environment have been reported [39,40,41,42]. It is an essential factor for plant growth as well as fruit products. During cucumber development, the environmental elements, especially solar radiation and temperature, were regarded as important factors [43,44]. However, research on the relationship between environment and cucumber fruit shape and size development is still lacking. 

In this study, a pair of NILs *Ln35* and *Ln37*, which carry gene *CsLNG* for fruit length QTL loci *FS2.1* in cucumber, were adopted to analyze the effects of genetic and environmental factors on cucumber fruit development. The results showed that *CsACS2* and *CsLNG* gene expression were the main internal factors that affected the different fruit shapes in this study between the two lines. The environmental factors were the main external factors that determined the fruit development and maturity period. The correlation analysis showed that the *CsACS2* and *CsLNG* gene expression were not significantly correlated with environmental factors. That is, the gene expression of *CsACS2* and *CsLNG* was not affected by environmental factors. This explains that the effect of environmental factors on fruit development and maturity period had almost no difference between *Ln35* and *Ln37*.

### 3.3. The Number of Days Needed for the Fruit of Commercial Maturity

The growing number of days needed for the fruit of commercial maturity might be forecasted according to the model. Usually, the average daily light intensity and average daily air temperature in local plastic tunnels were 24.73 °C and 11.86 Klux in the spring and 23.98 °C and 9.02 Klux in the autumn when it was suitable for the production of cucumber. It needed 9.63 day in the spring and 12.56 d in the autumn to reach the suitable length of commercial maturity fruits for *Ln35* and *Ln37*, respectively. It needed 10.69 d and 10.28 d in the spring and 13.96 d and 13.42 d in the autumn to reach the suitable diameter of commercial maturity fruits for *Ln35* and *Ln37*, respectively. It can provide precise management for round-shape cultivars and long-shape cultivars under different environmental conditions. 

Generally, cucumber fruits were consumed immaturely, and it needed different times for cucumber fruit from the ovary to grow into a commercial one in the spring and autumn. Based on the average fruit length and diameter at the commercial stage in two lines, it needed 12.47 MJ/m^2^ TEP in *Ln35* and 12.86 MJ/m^2^ TEP in *Ln37* for the fruit length to grow a suitable size from 0 dpa to the commercial stage. It needed 13.91 MJ/m TEP and 13.75 MJ/m^2^ TEP in *Ln37* for the fruit diameter to grow suitable size from 0 dpa to the commercial stage (Supplementary Table S3). In the application of a facility greenhouse, the temperature of cucumber fruit development is often set at 25–32 °C during the daytime and 20 °C at nighttime. For the light intensity, it needed 99.92 Klux in *Ln35* and 103.04 Klux in *Ln37* for the fruit length to grow suitable size from 0 dpa to commercial stage, and it needed 111.46 Klux and 110.18 Klux light intensity, respectively, for the fruit diameter to grow suitable size from 0 dpa to commercial stage. Under the natural light conditions, it needed 8.43 d and 8.69 d in spring and 9.78 d and 10.09 d in autumn to reach the suitable length of commercial maturity fruits for *Ln35* and *Ln37*, respectively. It needed 9.40 d and 9.29 d in the spring and 10.91 d and 10.78 d in the autumn to reach the suitable diameter of commercial maturity fruits for *Ln35* and *Ln37*, respectively. Supplemental lighting will shorter the time for the fruit to reach commercial maturity. Under the optimum temperature (25~32 °C at daytime and 20 °C at nighttime) and maximum light intensity (50 Klux) conditions for eight hours in the daytime, it only takes about 6 to 7 days for cucumber fruit growth to harvest stage. Therefore, in cucumber production, artificial heating or additional light supplementation can be used to promote the rate of cucumber fruit growth, shorten the fruit production cycle, and obtain greater economic value for the fruit diameter to grow a suitable size from 0 dpa to commercial stage.

## 4. Material and Methods

### 4.1. Plant Materials

A pair of near-isogenic lines (NILs), *Ln35* and *Ln37*, carrying gene *CsLNG* for fruit length QTL loci *FS2.1* in cucumber, were developed from a recombinant inbred lines population (RILs) [10,11,17], which were created by our research group. The *Ln35* carrying the wild-type *CsLNG* gene had a long cucumber shape with a fruit shape index of 3.48 at 12 days post anthesis (dpa), while *Ln37* carrying the mutant *cslng* gene had an ovate shape with a fruit shape index of 1.04 at 12 dpa. No significant difference between these two cucumber lines was found except for the fruit shape (Figure S1). 

### 4.2. Field Experiments

Two inbred cucumber lines, including *Ln35* and *Ln37*, were planted in two seasons. The seeds were sowed and cultured in substrate using 50-hole trays in a culture room, and seedlings with two fully spread true leaves were transplanted in a plastic tunnel at the Horticulture Experimental Station (34°16′ N, 108°4′ E) of the College of Horticulture, Northwest A&F University, Yangling, Shaanxi Province, China, 5 April 2020 in spring, and 4 August 2020 in autumn. A total of 3 replicates were grown for each inbred line, and 64 cucumber plants per replicate were included. Six rows were designed as the protection rows on both sides of the experiment site. The area of every plot was 4 × 2.4 m^2^ (length × width). The seedlings were planted on a plain rectangular pieces according to wide/narrow row alternation in which wide row spacing was 70 cm and narrow row spacing was 50 cm, and the plant spacing was 25 cm. One drip irrigation pipe was laid along the ridge before transplanting. A total of 64 seedlings were planted in each plot. A total of 10 m^3^ water per 667 m^2^ was irrigated to the plants from transplanting to recovering of seedlings. Fertilizers were applied five times with watering at the stage 10 days after transplanting, 20 days after transplanting, 50 days after transplanting, and 70 days after transplanting according to the growth characteristics of cucumber plants [45,46], which were shown in red font in the manuscript.

Two-time pre-experiments in the spring and autumn of 2019 were performed separately, in which we carefully observed the ovary size changes and the corresponding days before and after anthesis. Fruit sizes were measured from the beginning of the ovaries appearance until to the day of the fruit anthesis, and the corresponding days of fruit development were all recorded. The data were recorded and provided the reference for the formal statistics of fruit sizes before anthesis in 2020. The pre-experiment in 2019 provided us with a rather clear number of days about the approximate fruit sizes in autumn and spring 2020.

In the formal experiments, field experiments were conducted in two seasons, and one was performed from the early April to the late of June in 2020, and the other one was carried out from the early August to the late October in 2020. The data about fruit shape in spring were collected from the mid-May to the late of June, and which in autumn were collected from the early September to the late September. The data from the spring of 2020 were used as the model establishment and the data from the autumn of 2020 were used as the model verification.

To observe the fruit growth dynamics during the development, two hundred ovaries in *Ln35* or *Ln37* plants were marked once ovaries appeared at about the 14th node on 22 May 2020 and 3 September 2020. The ovaries with opening female flowers at the flowering day were selected and marked as 0 days post anthesis (0 dpa), and the ovaries with unopened female flowers before 0 dpa were selected and marked according to the number of days away from 0 dpa, which were recorded as −15, −12, − 9, −6, and −3 day pre-anthesis (−15, −12, −9, −6, and −3 dpa, respectively), and the fruits after the flowering day were selected and marked according to the number of days away from 0 day post anthesis were recorded as 3, 6, 9, 12, 15, and 18 days post anthesis (3, 6, 9, 12, 15, and 18 dpa, respectively). The fruit shape was measured every three days, along with the development of the fruit. When the fruits developed until the day of anthesis, the values of the fruit shape before anthesis were obtained. Excluding the data of fruits that did not flower on 6 June 2020 and 18 September 2020, the measurement and counting of the fruit shape after anthesis was continued to obtain the fruit shape data after flowering

The marked ovaries were measured carefully at 9:00~10:00 o’clock in the morning at −15, −12, −9, −6, −3, 0, 3, 6, 9, 12, 15, 18 dpa. The uniform and disease-free fruits were collected at 9:00 in the morning and transported to the laboratory immediately in the icebox. The fruits were divided into 3 parts: one part was stored at −80 °C, the other part was used for RNA extraction, and the third part was used for paraffin section analysis. 

### 4.3. Methods

#### 4.3.1. Data Collection about the Key Environmental Factors

The data of aerial temperature and light intensity were collected using the automatic environmental recorder (YM-18, Changmeng Electronic Science and Technology Limited Company, Handan, Hebei, China). The data were recorded automatically for every ten minutes. The aerial recorders were put on the place of the 2/3 plant height of cucumber plants. The daytime was defined from 07:00 to 19:00 and the nighttime was defined from 19:00 to 07:00 of the next day according to the local time of sunrise and sunset.

#### 4.3.2. Measurement of Fruit Length, Diameter and Volume

Fruit length and fruit diameter were used to describe the fruit’s shape and size. To observe the dynamic changes of fruit shape and size in NILs, cucumber fruits were measured at −15, −12, −9, −6, −3, 0, 3, 6, 9, 12, 15, and 18 dpa. The fruit length and diameter were measured using a digital caliper very carefully. The fruit shape index was the ratio of fruit length to diameter. The fruit length of the cucumber was the cumulative variable, which was the sum of fruit length elongation on the *i*-th day and the length on the *i*-1 of the previous day. As well as the fruit length and diameter were cumulative variables in cucumber. The fruit length increment and fruit diameter increment were adopted in this study, and every three days were calculated.

#### 4.3.3. Measurement of Cell Size during Cucumber Fruit Development

To observe the changes in fruit cell size during fruit development. Fruit cell length and fruit cell diameter were described as the fruit cell shape or size. The fruit longitudinal paraffin sections of *Ln35* and *Ln37* were prepared according to Guidarelli et al. [47]. The whole fruit of −9, −6, and −3 dpa, respectively, were used for embedding, and the fruits of 0, 3, 6, 12, 15, and 18 dpa were chopped into about 0.5 cm^2^ in the middle of the mesocarp and immediately fixed in 70% FAA solution. The tissues were then transferred into a graded ethanol series (10–100%) and chloroform series (10–100%) and embedded in paraffin. Then the samples were cut into 8 µm thick sections and, stained with safranine, sealed with gum. The sections were observed under an OLYMPUS BX51 microscope (OLYMPUS, Tokyo, Japan).

The cell size of fruits at different development stages was measured, and the cell density was counted in a 1 mm^2^ section using the software of Image J (National institutes of health, Bethesda, MD, USA). Five sections were selected randomly and measured. Five different fruits from different plants were used for every biological replication, and the experiment was repeated three times.

#### 4.3.4. qRT-PCR

The expression patterns of key genes related to the architecture of fruit size and shape in cucumbers were analyzed with quantitative real-time PCR. The primers of the six genes were provided in Supplementary Table S1. The six genes associated with the formation of fruit shape were chosen, including *CsSUN*, *CsLNG*, *CsFUL*, *CsSUP*, *CsACS2*, and *CsFNL*. RNA was extracted from the fruits of RILs at −9, −6, −3, 0, 3, 6, 12, 15, and 18 dpa using AG RNAex Pro Reagent (Accurate Biology, Hunan, China). The cDNA fragments were synthesized using the RNA by Transcriptor First Strand cDNA Synthesis Kit (Roche, Mannheim, Germany). The qRT-PCRs were performed using SYBR Green Master Mix (GeneStar, Beijing, China) with 20 μL reaction system on a QuantStudio5 (Life Technologies, Gaithersburg, MD, USA), and the data were analyzed using the 2^−ΔΔ^CT method and normalized by ubiquitin [48]. 

#### 4.3.5. Calculation of the Accumulated TEP

Thermal effectiveness and photosynthetic active radiation (TEP) were the product of thermal effectiveness (TE) and photosynthetic active radiation (PAR). TE was the accumulated value of relative thermal effectiveness (RTE), and RTE was proportional to actual temperature to optimal temperature under plant growth conditions. RTE was calculated according to Hang et al. [49] as follows:

(1) $$RTE(T)=\left\{\begin{array}{c} 0\\ \frac{T-T_{b}}{T_{ab}-T_{b}}\\ 1\\ \frac{T_{m}-T}{T_{m}-T_{ou}}\\ 0 \end{array}\right.\;\;\begin{array}{l} \left(T<T_{b}\right)\\ \left(T_{b}\leq T<T_{ab}\right)\\ \left(T_{ab}\leq T\leq T_{ou}\right)\\ \left(T_{ou}<T\leq T_{m}\right)\\ \left(T<T_{m}\right) \end{array}\;$$

where *T* corresponded to the mean of actual temperatures in one hour, *T_b_* was the lower limit temperature of the cucumber fruiting period, *T_ab_* was the lower limit of optimum temperature in the cucumber fruiting period, *T_m_* was the upper limit temperature, and *T_ou_* was the upper limit of optimum temperature in cucumber fruiting period. The *T_b_*, *T_ab_*, *T_ou,_* and *T_m_* temperatures in the cucumber fruiting period were defined at 16 °C, 25 °C, 32 °C, and 40 °C, respectively. 

PAR (MJ/(m^2^ × d) was the total solar radiation with a 0.5 photosynthesis coefficient in the solar light during one day [50]. The daily TEP (DTEP) was calculated according to Hang et al. [49] as follows the formula:

(2) DTEP(i)=((∑RTE(i,j))/24)×PAR(i)  (j=1,2,3…,24)

where *DTEP*(*i*) was the accumulated TEP on the *i*th day, *RTE*(*i*,*j*) was the RTE in the *j*th hour on the *i*th day, and *PAR*(*i*) was the PAR on the *i*th day. TEP (MJ/m^2^) was the accumulation of RET and PAR supply in the cucumber fruit growth period, which was calculated according to Formula (3):

(3) $$TEP=\sum_{i=1}^{N}DTEP(i)$$

In this study, TEP was calculated from −18 days pre-anthesis, which was regarded as the smallest fruit development stage visually and cannot be measured by vernier caliper.

#### 4.3.6. Statistical Analysis

Data from this study were subjected to statistical analysis, and significance tests were performed using one-way ANOVA analysis with Duncan’s multiple range test (*p* < 0.05). The significant difference in data was presented by an asterisk. Logistic regression was employed to analyze the relationship between the environmental TEP and fruit shape, including fruit length, diameter, and volume. The root mean squared error (RMSE) was conducted to analyze the conformity of the predicted value and measured value. RMSE values were calculated according to Nijssen [51] and Formula (4):

(4) $$RMSE=\sqrt{\frac{\sum_{i=1}^{n}{\left(OBSi-SIMi\right)}^{2}}{n}}$$

where *OBSi* was the measured value, *SIMi* was the predicted value, and *n* was the sample number. Linear regression was used to verify the models between the predicted value and measured value using 2020 autumn data. Model development and mathematical data analysis were performed by SPSS 22.0 (Statistical Package for the Social Sciences, Chicago, IL, USA) on Windows PC. The histograms were generated using Graph Pad Prism 6 (Graph Pad Software Inc., San Diego, CA, USA).

## 5. Conclusions

The study presented the relationship between fruit shape and size increment, cell size increment, and gene expression on the growth and development of a pair of *CsLNG* NIL cucumber lines. The correlations analysis revealed that cell number might contribute to the difference in fruit shape index between *Ln35* and *Ln37*, and *CsLNG* and *CsACS2* genes might act as the hereditary factors for the difference in cell size and fruit size between the two lines. The significant correlations between TE and fruit size and between RAC and fruit size indicated a potential capacity to apply mathematical models to predict cucumber fruit size development. Four models via logistic regression analysis were formulated, and the models were well validated using next year’s fruit size data and environmental data. 

## Figures and Tables

**Table 1:** The correlation analysis between gene expression, fruit increment, and cell increment.

Genes	Fruit Length Increment	Fruit Diameter Increment	Cell Length Increment	Cell Diameter Increment	Fruit Length Increment	Fruit Diameter Increment	Cell Length Inreament	Cell Diameter Increment
	*Ln35*				*Ln37*			
*CsACS2*	0.726 *	0.656 *	0.363	0.202	0.521	0.410	0.816 **	0.377
*CsFLU*	0.038	−0.028	−0.542	−0.473	−0.037	−0.131	0.076	−0.107
*CsSUN*	−0.323	−0.284	−0.353	−0.291	−0.324	−0.272	−0.178	−0.176
*CsFNL*	−0.328	−0.354	−0.453	−0.311	−0.277	−0.337	−0.291	−0.285
*CsLNG*	0.651 *	0.547	0.506	0.686 *	−0.243	−0.217	−0.198	−0.374
*CsSUP*	−0.549	−0.578	−0.450	−0.366	−0.451	−0.484	−0.278	−0.055

Asterisks indicate statistical difference of the values at *p* < 0.05 (*) or *p* < 0.01 (**).

**Table 2:** The correlation analysis between fruit shape and TE and PAR.

	Fruit Length	Fruit Diameter	Fruit Length	Fruit Diameter
	*Ln35*		*Ln37*	
TE	0.967 **	0.96 **	0.969 **	0.953 **
PAR	0.918 **	0.907 **	0.924 **	0.899 **

Asterisks indicate statistical difference of the values at *p* < 0.01 (**).

**Table 3:** Logistic regression equations of fruit shape for two inbred lines.

Fruit Traits		Modle	*R* ^2^	F	P	RMSE
Fruit length	*Ln35*	y = 1/(0.005 + 0.930 × 0.832^x^)	0.977	205.866	0	12.012
	*Ln37*	y = 1/(0.013 + 0.991 × 0.856^x^)	0.976	200.464	0	5.17
Fruit diameter	*Ln35*	y = 1/(0.016 + 1.384 × 0.863^x^)	0.960	117.786	0	4.338
	*Ln37*	y = 1/(0.014 + 1 × 0.859^x^)	0.987	422.541	0	7.082
Fruit volume	*Ln35*	y = 1/(0.002 + 522.51 × 0.708^x^)	0.981	261.447	0	43.07
	*Ln37*	y = 1/(0.004 + 593.002 × 0.712^x^)	0.987	418.87	0	19.19

## Data Availability

The raw data supporting the conclusions of this article will be made available by the authors, without undue reservation.

## Supplementary Materials

The following supporting information can be downloaded at: https://www.mdpi.com/article/10.3390/ijms232214033/s1.

## Abbreviations

TEP	thermal effectiveness and photosynthetic active radiation
QTLs	quantitative trait locus
Dpa	days post anthesis
NIL	near-isogenic line
TE	thermal effectiveness
PAR	photosynthetic active radiation
RMSE	root mean squared error

## References

[1] Ingrassia M., Sgroi F., Tudisca S., Chironi S. (2017). Study of Consumer Preferences in Regard to the Blonde Orange Cv. Washington Navel “Arancia Di Ribera PDO”. J. Food Prod. Mark..

[2] Rouphael Y., Kyriacou M.C., Petropoulos S.A., De Pascale S., Colla G. (2018). Improving vegetable quality in controlled environments. Sci. Hortic..

[3] Fanwoua J., de Visser P.H.B., Heuvelink E., Yin X., Struik P.C., Marcelis L.F.M. (2013). A dynamic model of tomato fruit growth integrating cell division, cell growth and endoreduplication. Funct. Plant Biol..

[4] Xu X., Wei C., Liu Q., Qu W., Qi X., Xu Q., Chen X. (2020). The major-effect quantitative trait locus *Fnl7.1* encodes a late embryogenesis abundant protein associated with fruit neck length in cucumber. Plant Biotechnol. J..

[5] Colle M., Weng Y., Kang Y., Ophir R., Sherman A., Grumet R. (2017). Variation in cucumber (Cucumis sativus L.) fruit size and shape results from multiple components acting pre-anthesis and post-pollination. Planta.

[6] Liu X., Pan Y., Liu C., Ding Y., Wang X., Cheng Z., Meng H. (2020). Cucumber Fruit Size and Shape Variations Explored from the Aspects of Morphology, Histology, and Endogenous Hormones. Plants.

[7] Marcelis L.F.M., Hofman-Eijer L.R.B. (1993). Cell division and expansion in the cucumber fruit. J. Hortic. Sci..

[8] Zhao J., Jiang L., Che G., Pan Y., Li Y., Hou Y., Zhao W., Zhong Y., Ding L., Yan S. (2019). A Functional Allele of *CsFUL1* Regulates Fruit Length through Repressing *CsSUP* and Inhibiting Auxin Transport in Cucumber. Plant Cell.

[9] Wang L., Cao C., Zheng S., Zhang H., Liu P., Ge Q., Li J., Ren Z. (2017). Transcriptomic analysis of short-fruit 1 (sf1) reveals new insights into the variation of fruit-related traits in Cucumis sativus. Sci. Rep..

[10] Pan Y., Liang X., Gao M., Liu H., Meng H., Weng Y., Cheng Z. (2017). Round fruit shape in WI7239 cucumber is controlled by two interacting quantitative trait loci with one putatively encoding a tomato SUN homolog. Theor. Appl. Genet..

[11] Wu S., Zhang B., Keyhaninejad N., Rodríguez G.R., Kim H.J., Chakrabarti M., Illa-Berenguer E., Taitano N.K., Gonzalo M.J., Díaz A. (2018). A common genetic mechanism underlies morphological diversity in fruits and other plant organs. Nat. Commun..

[12] Che G., Zhang X. (2019). Molecular basis of cucumber fruit domestication. Curr. Opin. Plant Biol..

[13] Xin T., Zhang Z., Li S., Zhang S., Li Q., Zhang Z.-H., Huang S., Yang X. (2019). Genetic Regulation of Ethylene Dosage for Cucumber Fruit Elongation. Plant Cell.

[14] Gao Z., Zhang H., Cao C., Han J., Li H., Ren Z. (2020). QTL Mapping for Cucumber Fruit Size and Shape with Populations from Long and Round Fruited Inbred Lines. Hortic. Plant J..

[15] Wei Q., Wang Y., Qin X., Zhang Y., Zhang Z., Wang J., Li J., Lou Q., Chen J. (2014). An SNP-based saturated genetic map and QTL analysis of fruit-related traits in cucumber using specific-length amplified fragment (SLAF) sequencing. BMC Genom..

[16] Weng Y., Colle M., Wang Y., Yang L., Rubinstein M., Sherman A., Ophir R., Grumet R. (2015). QTL mapping in multiple populations and development stages reveals dynamic quantitative trait loci for fruit size in cucumbers of different market classes. Theor. Appl. Genet..

[17] Zhang T., Li X., Yang Y., Guo X., Feng Q., Dong X., Chen S. (2019). Genetic analysis and QTL mapping of fruit length and diameter in a cucumber (*Cucumber sativus* L.) recombinant inbred line (RIL) population. Sci. Hortic..

[18] Kami C., Lorrain S., Hornitschek P., Fankhauser C. (2010). Light-Regulated Plant Growth and Development. Curr. Top. Dev. Biol..

[19] Léchaudel M., Joas J. (2007). An overview of preharvest factors influencing mango fruit growth, quality and postharvest behaviour. Braz. J. Plant Physiol..

[20] Warrington I., Fulton T., Halligan E., De Silva H. (1999). Apple Fruit Growth and Maturity are Affected by Early Season Temperatures. J. Am. Soc. Hortic. Sci..

[21] Ali A., Kelly W. (1993). Effect of pre-anthesis temperature on the size and shape of sweet pepper (*Capsicum annuum* L.) fruit. Sci. Hortic..

[22] Dorais M., Gosselin A., Trudel M.J. (1991). Annual greenhouse tomato production under a sequential intercropping system using supplemental light. Sci. Hortic..

[23] Uzun S. (2007). Effect of light and temperature on the phenology and maturation of the fruit of eggplant (*Solanum melongena*) grown in greenhouses. N. Z. J. Crop Hortic. Sci..

[24] Gómez C., Izzo L.G. (2018). Increasing efficiency of crop production with LEDs. AIMS Agric. Food.

[25] Palmitessa O., Pantaleo M., Santamaria P. (2021). Applications and Development of LEDs as Supplementary Lighting for Tomato at Different Latitudes. Agronomy.

[26] Zhou T.-M., Wu Z., Wang Y.-C., Su X.-J., Qin C.-X., Huo H.-Q., Jiang F.-L. (2019). Modelling seedling development using thermal effectiveness and photosynthetically active radiation. J. Integr. Agric..

[27] Boudon F., Persello S., Jestin A., Briand A.-S., Grechi I., Fernique P., Guédon Y., Léchaudel M., Lauri P., Normand F. (2020). V-Mango: A functional–structural model of mango tree growth, development and fruit production. Ann. Bot..

[28] Fishman S., Génard M. (2010). Biophysical model of fruit growth: Simulation of seasonal and diurnal dynamics of mass. Plant Cell Environ..

[29] Lescourret F., Génard M., Habib R., Pailly O. (1998). Pollination and fruit growth models for studying the management of kiwifruit orchards. II. Models behaviour. Agric. Syst..

[30] Bepete M., Lakso A. (1997). Apple fruit respiration in the field: Relationships to fruit growth rate, temperature, and light exposure. Acta Hortic..

[31] Riga P. (2015). Effect of rootstock on growth, fruit production and quality of tomato plants grown under low temperature and light conditions. Hortic. Environ. Biotechnol..

[32] Chang L.-Y., He S.-P., Liu Q., Xiang J.-L., Huang D.-F. (2018). Quantifying muskmelon fruit attributes with A-TEP-based model and machine vision measurement. J. Integr. Agric..

[33] Li Y., Luo W., Ni J., Chen Y., Xu G., Jin L., Dai J., Chen C. (2005). Simulation of leaf area, photosynthetic rate and dry matter production in greenhouse cucumber based on product of thermal effectiveness and photosynthetically active radiation. Trans. CSAE.

[34] Ando K., Grumet R. (2010). Transcriptional Profiling of Rapidly Growing Cucumber Fruit by 454-Pyrosequencing Analysis. J. Am. Soc. Hortic. Sci..

[35] Fu F.Q., Mao W.H., Shi K., Zhou Y.H., Asami T., Yu J.Q. (2008). A role of brassinosteroids in early fruit development in cucumber. J. Exp. Bot..

[36] Yang X.Y., Wang Y., Jiang W.J., Liu X.L., Zhang X.M., Yu H.J., Huang S.W., Liu G.Q. (2013). Characterization and expression profiling of cucumber kinesin genes during early fruit development: Revealing the roles of kinesins in exponential cell production and enlargement in cucumber fruit. J. Exp. Bot..

[37] Dahan Y., Rosenfeld R., Zadiranov V., Irihimovitch V. (2010). A proposed conserved role for an avocado fw2.2-like gene as a negative regulator of fruit cell division. Planta.

[38] Corelli L., Lakso A.N. (2004). Fruit development in deciduous tree crops as affected by physiological factors and environmental conditions. Acta Hortic..

[39] Zamljen T., Zupanc V., Slatnar A. (2020). Influence of irrigation on yield and primary and secondary metabolites in two chilies species, *Capsicum annuum* L. and *Capsicum chinense* Jacq. Agric. Water Manag..

[40] Chen G., Wiatrak P. (2010). Soybean Development and Yield Are Influenced by Planting Date and Environmental Conditions in the Southeastern Coastal Plain, United States. Agron. J..

[41] Kim H.J., Jung H.H., Kim K.S. (2011). Influence of photoperiod on growth and flowering of dwarf purple loosestrife. Hortic. Environ. Biotechnol..

[42] Rajasekar M., Arumugam T., Kumar S. (2013). Influence of weather and growing environment on vegetable growth and yield. J. Hortic. For..

[43] Alsadon A., Al-Helal I., Ibrahim A., Abdel-Ghany A., Al-Zaharani S., Ashour T. (2016). The effects of plastic greenhouse covering on cucumber (*Cucumis sativus* L.) growth. Ecol. Eng..

[44] Hao X., Papadopoulos A.P. (1999). Effects of supplemental lighting and cover materials on growth, photosynthesis, biomass partitioning, early yield and quality of greenhouse cucumber. Sci. Hortic..

[45] Beyaert R.P., Roy R.C., Coelho B.R.B. (2007). Irrigation and fertilizer management effects on processing cucumber productivity and water use efficiency. Can. J. Plant Sci..

[46] Souri M.K., Sooraki F.Y., Moghadamyar M. (2017). Growth and quality of cucumber, tomato, and green bean under foliar and soil applications of an aminochelate fertilizer. Hortic. Environ. Biotechnol..

[47] Guidarelli M., Carbone F., Mourgues F., Perrotta G., Rosati C., Bertolini P., Baraldi E. (2011). Colletotrichum acutatum interactions with unripe and ripe strawberry fruits and differential responses at histological and transcriptional levels. Plant Pathol..

[48] Livak K.J., Schmittgen T.D. (2001). Analysis of relative gene expression data using real-time quantitative PCR and the 2^−ΔΔCT^ Method. Methods.

[49] Hang T., Lu N., Takagaki M., Mao H. (2019). Leaf area model based on thermal effectiveness and photosynthetically active radiation in lettuce grown in mini-plant factories under different light cycles. Sci. Hortic..

[50] Xiangxiang W., QuanJiu W., Jun F., Lijun S., Xinlei S. (2014). Logistic model analysis of winter wheat growth on China’s Loess Plateau. Can. J. Plant Sci..

[51] Nijssen B., O’Donnell G.M., Lettenmaier D.P., Lohmann D., Wood E. (2001). Predicting the Discharge of Global Rivers. J. Clim..

