# Supplementary figures and images for: Relationship between Key Environmental Factors and the Architecture of Fruit Shape and Size in Near-Isogenic Lines of Cucumber (Cucumis sativus L.)
Source: Int J Mol Sci. 2022 Nov 14;23(22):14033. doi: 10.3390/ijms232214033 (PMC9697376; doi:10.3390/ijms232214033)

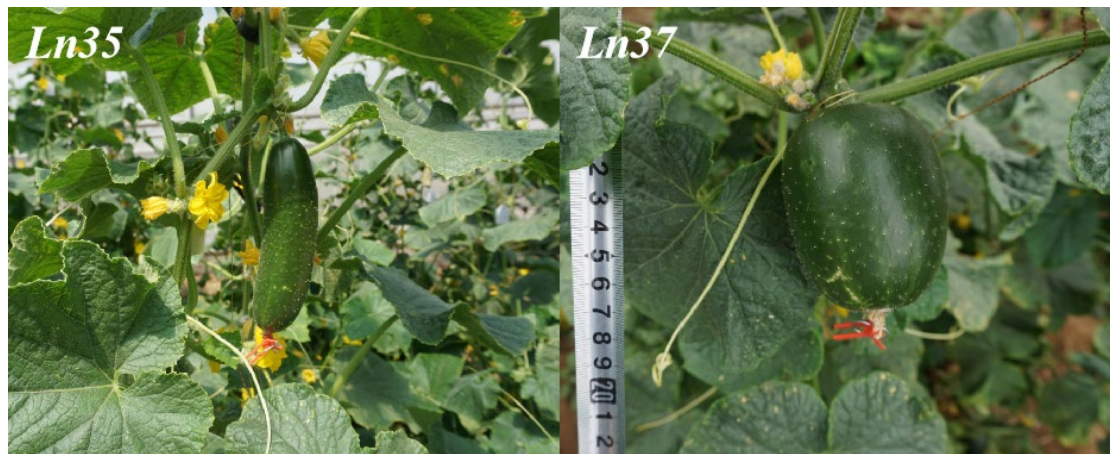

**Figure S1.** Morphological observation of fruit size and shape in *Ln35* and *Ln37*.

Supplement: Supplementary file 1 [file ijms-23-14033-s001.zip › Supplemental figure.pdf]
